# Interventional treatment of a bacterial embolus in the retrohepatic inferior vena cava secondary to invasive Klebsiella pneumoniae liver abscess syndrome: a case report and literature review

**DOI:** 10.3389/fcvm.2026.1923015

**Published:** 2026-08-06

**Authors:** Yun-Dong Chen, Qi Dong, Juan Li, Da-Wen Cheng

**Affiliations:** 1 Department of Interventional Radiology, Nanjing Hospital of Chinese Medicine Affiliated to Nanjing University of Chinese Medicine, Nanjing, China; 2 Department of Otolaryngology Head and Neck Surgery, Affiliated Drum Tower Hospital of Nanjing University Medical School, Jiangsu Provincial Key Medical Discipline (Laboratory), Nanjing, China

**Keywords:** bacterial embolus, case report, inferior vena cava, interventional radiology, invasive Klebsiella pneumoniae liver abscess syndrome

## Abstract

**Background:**

Retrohepatic inferior vena cava (IVC) bacterial embolism secondary to invasive Klebsiella pneumoniae liver abscess syndrome (IKPLAS) is extremely rare, and no English literature has reported its interventional treatment to date. This study presents a detailed case of such interventional treatment, providing references for clinical practice.

**Case presentation:**

A 53-year-old female with a 1-year history of type 2 diabetes presented with fever. Laboratory tests showed elevated inflammatory markers, blood culture confirmed pan-sensitive K. pneumoniae, and abdominal ultrasound detected a liver abscess, leading to the diagnosis of IKPLAS. Doppler ultrasound revealed hepatic vein thrombi and a suspected retrohepatic IVC thrombus. She was transferred to our hospital for further management. At our hospital, ultrasound-guided percutaneous catheter drainage of the abscess was performed (pus culture confirmed K. pneumoniae). Subsequently, interventional treatment was performed: an 18 mm × 60 mm PTA dilatation catheter was inflated to prevent bacterial embolus detachment; then a 10F peripheral thrombectomy catheter was used for repeated aspiration, successfully retrieving white massive/filamentous bacterial emboli. Final venography showed complete resolution of the IVC filling defect, restored patency, and no perioperative complications. The patient received a total of 6 weeks of antimicrobial therapy and 3 months of oral rivaroxaban post-discharge. Follow-up at 6 months and 1 year showed complete resolution of all lesions with no recurrence.

**Conclusion:**

We describe a case of successful interventional treatment (balloon dilation combined with catheter aspiration thrombectomy) for this condition. Interventional therapy may be an effective adjunct to antibiotic and anticoagulation therapy for IKPLAS-related IVC bacterial embolism.

## 1 Introduction

Approximately 20% of patients with Klebsiella pneumoniae (K. pneumoniae) liver abscess develop extrahepatic infections, a condition defined as invasive K. pneumoniae liver abscess syndrome (IKPLAS) (1). The most common extrahepatic sites include the lungs, eyes, central nervous system (CNS), and soft tissues, which may progress to severe complications such as empyema, endophthalmitis, meningitis, brain abscess, and necrotizing fasciitis. Rare complications (e.g., prostatic abscesses, infective endocarditis) have also been reported, reflecting the syndrome's heterogeneous clinical spectrum (2–4).

Epidemiologically, IKPLAS was first described in Taiwan, China, in the 1980s (5). Since then, cases have increased across Asia, with incidence having risen in North America; European reports remain scarce (6). Clinically, IKPLAS is characterized by abrupt onset, rapid progression, and nonspecific presentations, leading to high morbidity and mortality, as well as challenges in clinical management—thus drawing growing attention from the medical community. A systematic search was conducted across five databases (PubMed, EMbase, The Cochrane Library, Web of Science, and Scopus) with a cutoff date of July 17, 2026, using the search query: (Klebsiella pneumoniae) AND (Inferior Vena Cava). The initial search retrieved 87 records in total: 6 from PubMed, 43 from EMbase, 1 from The Cochrane Library, 6 from Web of Science, and 31 from Scopus. After removing duplicate records, 50 unique citations underwent full-text evaluation. Ultimately, a total of 8 previously reported cases of Klebsiella pneumoniae bacteremia complicated by IVC embolism were included. Among them, 7 cases were secondary to liver abscess, and 1 case occurred without concomitant liver abscess. All previously documented cases were managed with systemic antimicrobial therapy combined with anticoagulation, and all achieved clinical improvement. No prior report has described progressive IVC bacterial embolism requiring endovascular interventional treatment after standard medical therapy.

## 2 Case report

A 53-year-old female patient (body weight: 58 kg) presented to a local hospital with fever, with the peak temperature reaching 40 °C. Laboratory tests yielded the following results: white blood cell (WBC) count: 12.56 × 10^9^/L; neutrophil percentage (N%): 85.9%; C-reactive protein (CRP): 308.5 mg/L; procalcitonin (PCT): 0.44 ng/mL; interleukin-6 (IL-6): 148.5 pg/mL; alkaline phosphatase (ALP): 263 U/L; alanine aminotransferase (ALT): 14.3 U/L; aspartate aminotransferase (AST): 17.2 U/L. Blood culture identified pan-sensitive K. pneumoniae. During hospitalization, the patient developed severe pneumonia and bilateral pleural effusion, and bilateral thoracic drainage was performed accordingly. The pleural effusion was described as serous and clear via follow-up inquiry, but the microbiological culture results from the local hospital were not available for review after transfer, so it remains unclear whether these pulmonary manifestations represent metastatic infectious complications of IKPLAS. Abdominal ultrasound detected a liver abscess, and IKPLAS was initially suspected based on K. pneumoniae bacteremia and liver abscess with extrahepatic involvement. Her medical history included type 2 diabetes mellitus (T2DM) for 1 year, previously complicated by diabetic ketoacidosis, and she required insulin therapy (basal-bolus regimen). The patient denied a history of smoking or alcohol consumption.

Initially, the local hospital administered antimicrobial therapy with intravenous cefoxitin (2 g every 8 h). After 7 days, recurrent fevers (38.5 °C–39.5 °C) were observed, and repeat blood cultures remained positive for the identical K. pneumoniae strain. Based on these findings, the antimicrobial regimen was escalated to intravenous imipenem-cilastatin (1 g every 8 h). Following this adjustment, the patient's temperature decreased and stabilized at approximately 37.5 °C. However, follow-up Doppler ultrasound revealed thrombi within the middle and right hepatic veins, with a suspected thrombus adherent to the retrohepatic IVC. Low-molecular-weight heparin (enoxaparin 4,000 U subcutaneously every 12 h) was promptly initiated, and the patient was subsequently referred to our hospital for further management.

**Figure 1:**
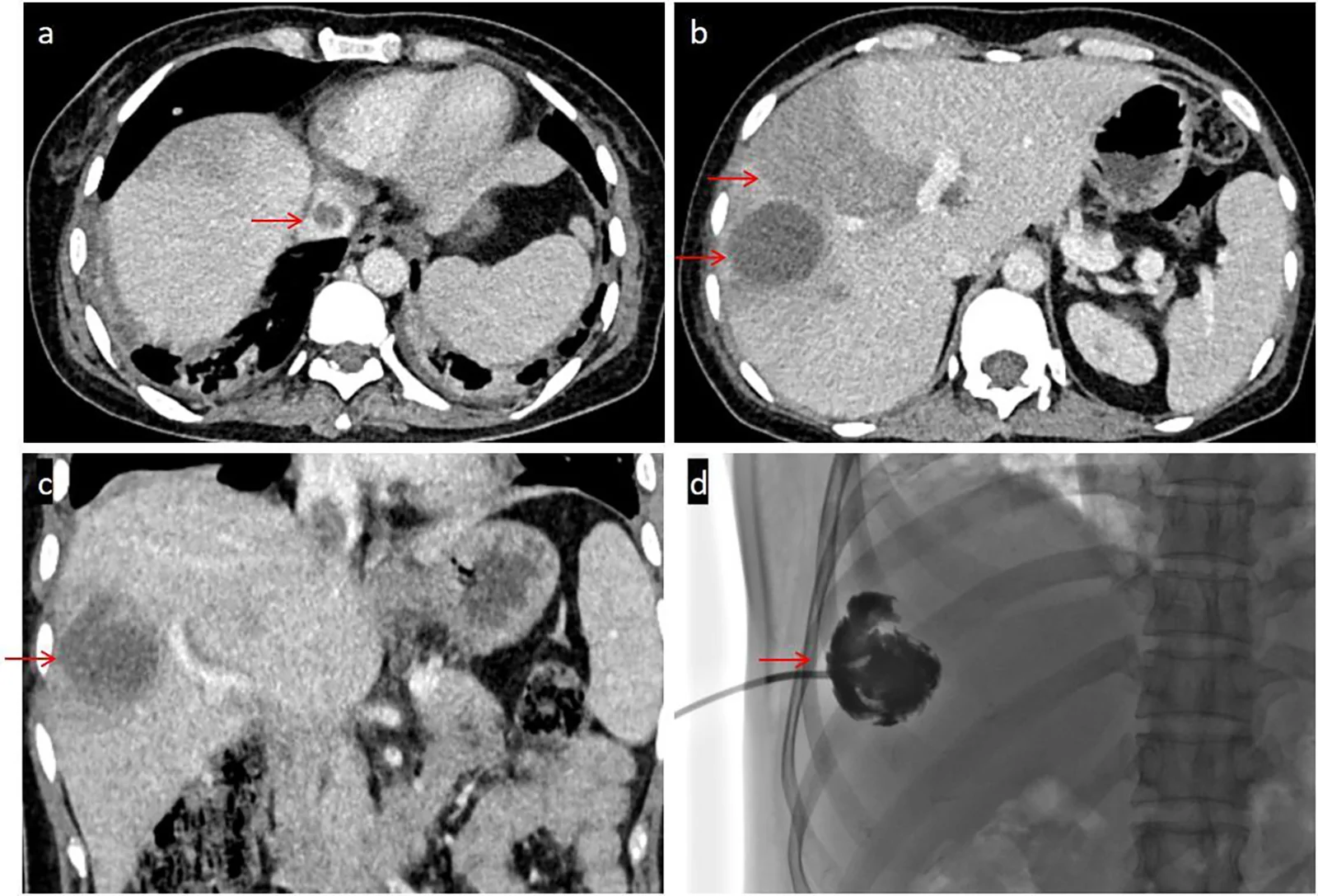
Upper abdominal contrast-enhanced CT and percutaneous liver abscess puncture drainage. **(a)** Revealed a bacterial embolus in the retrohepatic IVC. **(b,c)** Showed thrombosis in the right and middle hepatic veins, accompanied by hepatic ischemia and infarction in the drainage territory of the middle hepatic vein; additionally, a liver abscess was also identified in the corresponding hepatic region. **(d)** Showed the performance of percutaneous liver abscess puncture drainage.

Upon admission to our hospital (on the 9th day of anticoagulation therapy), baseline laboratory tests showed an estimated glomerular filtration rate (eGFR) of 106 mL/min/1.73 m^2^ and glycated hemoglobin A1c (HbA1c) of 8.52%, indicating preserved renal function and poor long-term glycemic control. The patient subsequently underwent contrast-enhanced abdominal computed tomography (CT), which demonstrated a 25.3 mm × 16.8 mm × 12.7 mm embolism in the retrohepatic IVC causing approximately 73% luminal stenosis. Additional findings included thrombosis in the right and middle hepatic veins, ischemic hepatic infarction in the drainage territory of the middle hepatic vein, and a residual 44.9 mm × 34.9 mm liver abscess containing gas locules (Figures 1a–c). The patient denied any ocular discomfort or neurological symptoms such as headache or altered consciousness, so specialized ophthalmologic evaluation and central nervous system assessment were not performed. Transthoracic echocardiography was completed, revealing mild mitral regurgitation, mild tricuspid regurgitation, and left ventricular diastolic dysfunction, with no evidence of valvular vegetations indicative of infective endocarditis. Under ultrasound guidance, percutaneous catheter drainage of the liver abscess was performed, yielding turbid yellow pus, after which an 8F drainage catheter (Boston Scientific, USA) was placed (Figure 1d). Pus culture further confirmed growth of K. pneumoniae, consistent with the previous isolate.

**Figure 2:**
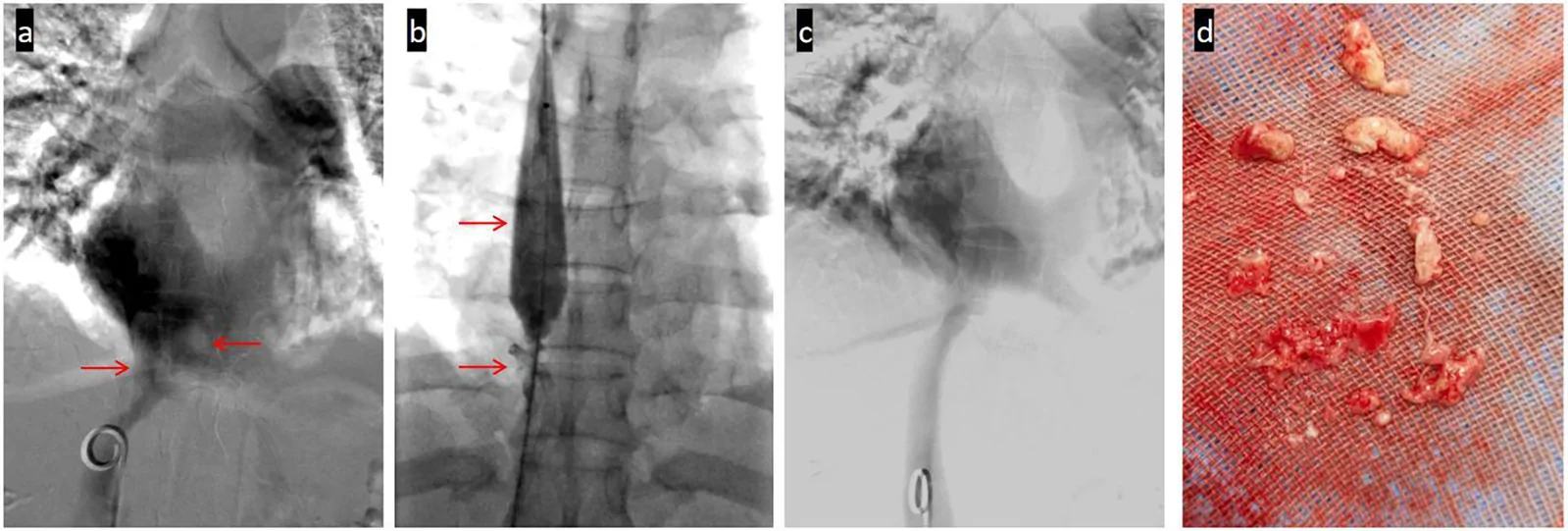
Interventional treatment for IVC bacterial embolus. **(a)** Showed massive filling defects in the retrohepatic IVC. **(b)** Demonstrated balloon positioning above the bacterial embolus and placement of the peripheral thrombectomy catheter for aspiration. **(c)** Postoperative cavography demonstrated complete resolution of the IVC filling defect and restoration of luminal patency. **(d)** Showed the retrieved white massive and filamentous bacterial embolus.

Given the large bacterial embolus burden, severe IVC luminal stenosis, progressive disease despite 8 days of combined antibiotic and anticoagulation therapy, compromised hepatic venous outflow, and high risk of septic pulmonary embolism, the multidisciplinary clinical team decided to perform endovascular aspiration thrombectomy to rapidly remove the infective embolic material and restore venous patency. Subsequently, interventional treatment was performed to address the retrohepatic IVC bacterial embolus. The procedure was performed under local infiltration anesthesia without intravenous sedation, with a total procedural time of 70 min. Systemic heparinization with 4,000 units of unfractionated heparin was administered at the onset of the procedure, and 10F vascular sheaths were placed in both femoral veins. First, a 5F PIG catheter was advanced via the right femoral access into the distal IVC, and venography confirmed massive filling defects in the retrohepatic IVC (Figure 2a). Next, a 0.035-inch guidewire was carefully advanced across the bacterial embolus into the right internal jugular vein via the left femoral access to establish a translesional pathway. Given the close proximity of the embolus to the right atrium and insufficient proximal venous length for safe IVC filter deployment, balloon protection was selected as the preferred embolic prophylaxis strategy. Over this guidewire, a MAXI 18 mm × 60 mm PTA dilatation catheter (Cordis, USA) was positioned above the embolism and gradually inflated using a pressure pump, serving as proximal protection against embolus dislodgement(Figure 2b). During balloon inflation, the patient's vital signs were closely monitored: heart rate increased from 110 to 120 beats per minute, while blood pressure, respiratory rate, and oxygen saturation remained stable, and the patient reported no symptoms such as chest pain or dyspnea. Following balloon dilation, a 10F peripheral thrombectomy catheter (Acotec, China) was advanced via the right femoral access to the site of the retrohepatic IVC bacterial embolus, and a total of 4 aspiration passes were performed (Figure 2b). The total balloon inflation duration was 9 min. White massive and filamentous bacterial emboli were successfully retrieved during the procedure (Figure 2d). Total estimated intraoperative blood loss was approximately 50 mL, and total contrast volume was less than 100 mL. Final venography showed complete resolution of the filling defect in the retrohepatic IVC, restored luminal patency, and brisk flow through the IVC (Figure 2c). The interventional procedure was concluded without any perioperative complications.

**Figure 3:**
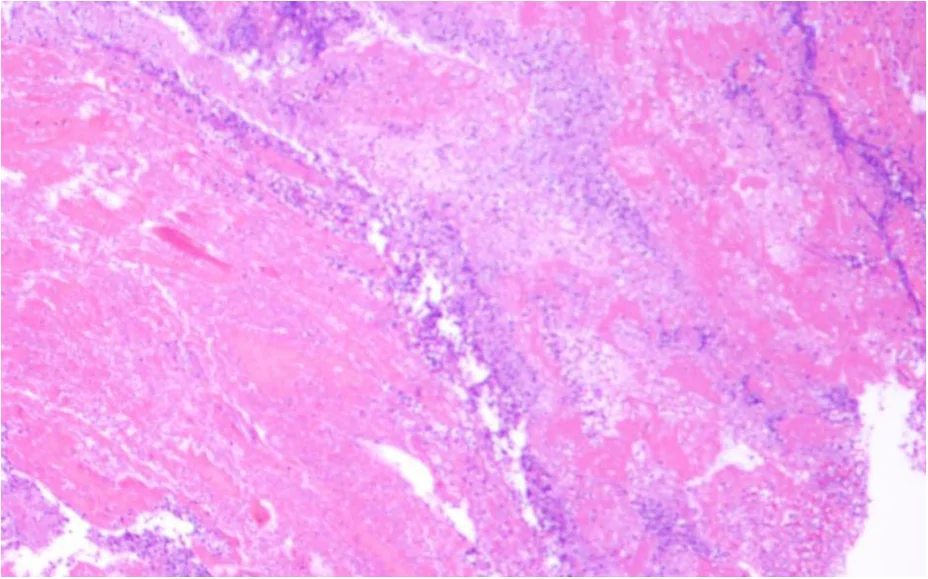
Histopathological examination of aspirated bacterial embolus. The aspirated bacterial emboli were composed of blood clots and inflammatory necrotic tissue.

Histopathological examination of the aspirated bacterial embolus revealed the presence of abundant inflammatory necrotic debris admixed with blood clots (Figure 3). Culture of the bacterial embolus demonstrated the growth of K. pneumoniae, which thereby confirmed the infectious nature of the bacterial embolus. On postoperative day 8, the liver abscess drainage catheter was removed, as the drainage output was persistently minimal and follow-up CT showed significant resolution of the liver abscess.

**Figure 4:**
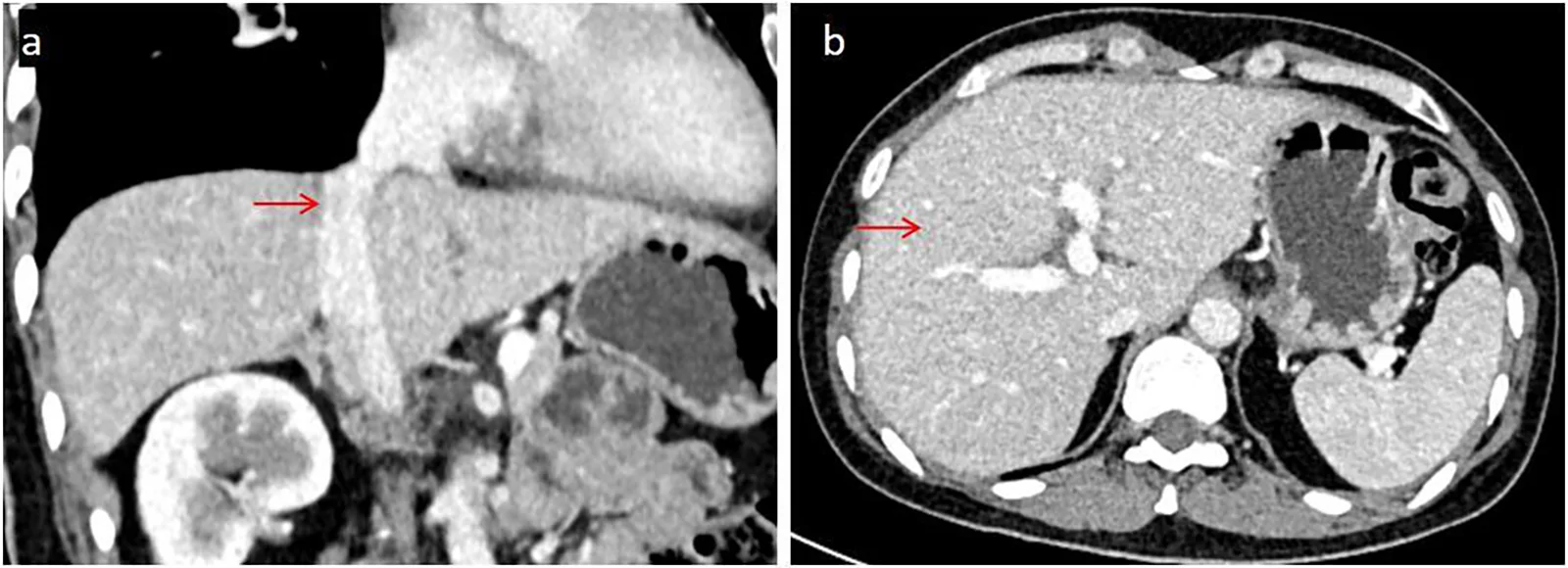
Follow-up upper abdominal contrast-enhanced CT at 6 months after discharge. **(a)** demonstrated the complete resolution of the IVC bacterial embolus; **(b)** demonstrated the complete resolution of the hepatic abscess and hepatic ischemic infarct lesions.

During hospitalization, the patient received a 4-week course of intravenous antimicrobial therapy. Following sustained defervescence and marked reduction in inflammatory markers, therapy was de-escalated to piperacillin-sulbactam to minimize unnecessary carbapenem exposure and reduce selective pressure for resistant *Enterobacterales*. The 2:1 piperacillin:sulbactam formulation was dosed at 6 g (4 g piperacillin + 2 g sulbactam) intravenously every 12 h. Anticoagulation therapy was maintained concurrently throughout the treatment period. No recurrence of fever was observed thereafter. Repeat contrast-enhanced abdominal CT showed complete resolution of the IVC bacterial embolus and improvement in hepatic ischemic infarction. The total duration of antimicrobial therapy for the patient, combining that at the local hospital and our hospital, was 6 weeks. At discharge, laboratory findings revealed normalized inflammatory markers, including a WBC count of 5.9 × 10^9^/L, CRP < 1 mg/L, and PCT 0.03 ng/mL. Multiple sets of blood cultures were negative for pathogenic growth. The patient was discharged on oral rivaroxaban 20 mg once daily for a total of 3 months of anticoagulation. Follow-up abdominal CT examinations at 6 months and 1 year after discharge respectively confirmed complete resolution of both the liver abscess and hepatic ischemic infarction, with no evidence of disease recurrence (Figure 4). The full diagnostic and therapeutic course of this case is summarized in a chronological timeline for clearer presentation (see Table 1).

**Table 1:** Timeline of diagnosis and treatment for the patient with retrohepatic IVC bacterial embolus secondary to IKPLAS.

Time point	Diagnostic/therapeutic measures	Key outcomes
Day 1 (local hospital)	Admitted due to fever (peak temperature: 40 °C); Completed complete blood count (CBC) and blood culture; Initiated intravenous cefoxitin (2 g every 8 h) for anti-infection therapy	CBC showed white blood cell (WBC) count: 12.56 × 10^9^/L, C-reactive protein (CRP): 308.5 mg/L; fever persisted
Day 3 (local hospital)	Completed chest computed tomography (CT) and abdominal ultrasound; Obtained blood culture results	Blood culture was positive for pan-sensitive Klebsiella pneumoniae; Chest CT indicated severe pneumonia with bilateral pleural effusion, and abdominal ultrasound detected a liver abscess; Diagnosed as IKPLAS based on fever symptoms, laboratory findings, and imaging results
Day 8 (local hospital)	Adjusted antimicrobial regimen to intravenous imipenem-cilastatin (1 g every 8 h); Repeated abdominal ultrasound; Initiated anticoagulation therapy with low-molecular-weight heparin (enoxaparin 4,000 U subcutaneously every 12 h)	Repeated abdominal ultrasound revealed thrombosis in the hepatic veins and retrohepatic IVC
Day 16 (our hospital)	Completed contrast-enhanced abdominal CT; Underwent ultrasound-guided percutaneous catheter drainage of the liver abscess, with placement of an 8F drainage catheter	Contrast-enhanced abdominal CT confirmed retrohepatic IVC embolism (73% luminal stenosis); Pus culture from the abscess was positive for Klebsiella pneumoniae (consistent with the blood culture isolate)
Day 18 (our hospital, interventional therapy day)	Underwent retrohepatic IVC interventional therapy: 18 mm × 60 mm PTA dilatation catheter inflated to prevent bacterial embolus dislodgement and subsequent pulmonary embolism; 10F peripheral thrombectomy catheter used for aspiration	Postoperative venography showed patent retrohepatic IVC without filling defects; No perioperative complications (e.g., bleeding, pulmonary embolism)
Day 40 (our hospital)	Repeated contrast-enhanced upper abdominal CT	Complete resolution of retrohepatic IVC bacterial embolus; Improved ischemic hepatic infarction in the territory of the middle hepatic vein; Reduced liver abscess size
Day 42 (our hospital)	Repeated inflammatory markers [WBC, CRP, procalcitonin (PCT)]; Discontinued antimicrobial therapy	Inflammatory markers returned to normal: WBC 5.9 × 10^9^/L, CRP < 1 mg/L, PCT 0.03 ng/mL
Day 45 (our hospital, total hospital stay: 30 days)	Discharged from hospital	Total duration of antimicrobial therapy: 6 weeks (2 weeks at local hospital + 4 weeks at our hospital); Discharge medication: oral rivaroxaban (20 mg once daily) for 3-month anticoagulation
Post-discharge (6 months and 1 year)	Follow-up contrast-enhanced abdominal CT	Abdominal CT confirmed complete resolution of liver abscess and ischemic hepatic infarction; no evidence of disease recurrence

## 3 Discussion

K. pneumoniae is a facultatively anaerobic Gram-negative bacillus and a typical opportunistic pathogen associated with pneumonia, urinary tract infections, and bacteremia, particularly in immunocompromised hosts. In contrast, IKPLAS is predominantly caused by hypervirulent K. pneumoniae (hvKP) (7). Unlike classical K. pneumoniae (cKp), hvKP frequently infects otherwise healthy individuals (8) and can colonize the gastrointestinal tract, facilitating fecal-oral transmission in both community and healthcare settings—a major challenge for infection control.

Notably, specialized microbiological assays for definitive hvKP confirmation—including the string test for hypermucoviscosity phenotype, capsular serotyping, virulence gene detection, and whole-genome analysis—were not performed in this case. Accordingly, the diagnosis of IKPLAS in this patient was established on clinical grounds, based on the constellation of K. pneumoniae bacteremia, pyogenic liver abscess, and metastatic extrahepatic infectious complications, rather than on microbiologically confirmed hvKP infection.

Phenotypically, hvKP often exhibits a hypermucoviscous appearance, classically identified by a positive “string test,” defined as production of a viscous string ≥5 mm when a colony is stretched with a loop or needle (9). Consequently, these strains are sometimes termed hypermucoviscous (HMV) K. pneumoniae. However, HMV phenotype does not strictly correlate with IKPLAS: not all IKPLAS isolates are HMV-positive, and not all HMV strains cause IKPLAS.

Molecular mechanisms underlying hvKP long-distance dissemination involve synergistic actions of capsular polysaccharides (CPS), iron-acquisition systems, and key virulence genes (7). Genes such as iucA, iroB, peg-344, rmpA, and rmpA2 can regulate siderophore production and mucus viscosity, contributing to enhanced virulence (10). Polymerase chain reaction (PCR)-based detection of virulence genes [e.g., rmpA (Regulator of Mucoid Phenotype A), magA (Mucoviscosity-Associated Gene A)] offers precise identification of hvKP, improving diagnostic accuracy (3).

Serotyping provides another dimension for understanding pathogenicity. K. pneumoniae has 78 known capsular serotypes, among which K1 and K2 are strongly associated with IKPLAS (7). These serotypes evade neutrophil-mediated killing, promoting hematogenous spread. However, some studies suggest no significant difference in HMV phenotype or serotype distribution across different K. pneumoniae groups (1). These discrepancies in research conclusions suggest that the pathogenesis of IKPLAS may be more complex, and a single phenotype or serotype indicator is insufficient for comprehensive and accurate diagnosis of the disease. Therefore, in clinical practice, the diagnosis of IKPLAS largely relies on a comprehensive assessment of the patient's clinical manifestations rather than solely on microbiological markers—consistent with the diagnostic approach applied in the present case.

HvKP infection is associated with thromboembolic complications, as it can trigger disruption of vascular integrity and exacerbate disease severity. The systemic inflammation induced by hvKP exerts procoagulant effects, and its capsule and lipopolysaccharide (LPS) directly activate endothelial cells, promote tissue factor upregulation and enhance platelet aggregation (11). In addition, hvKP may induce the formation of neutrophil extracellular traps (NETs), which form a matrix that captures platelets and fibrin, thereby contributing to thrombus formation (12).

Key risk factors for IKPLAS include Asian ethnicity, diabetes mellitus, prior antibiotic exposure, obesity, advanced age, and alcohol use (13). Diabetes plays a pivotal role: hyperglycemia provides a nutrient-rich environment for bacterial proliferation, while impaired immunity facilitates systemic dissemination, including penetration of the blood-brain and blood-eye barriers (14). Mechanistically, neutrophil extracellular traps (NETs) are critical in combating hvKP. However, NET-mediated bacterial killing is impaired in T2DM patients—not due to reduced NETs formation, but due to insufficient ability of NETs to damage the surface of pathogens (15). Our patient's poor glycemic control (HbA1c 8.52%) was consistent with the aforementioned mechanism. Additionally, the use of ampicillin/amoxicillin within the past 30 days may promote intestinal overgrowth of K. pneumoniae, increasing the risk of IKPLAS (16). However, our patient denied such exposure.

Other independent predictors of IKPLAS include liver abscess diameter ≤5.8 cm and elevated serum lactate levels (17, 18). Early bacteremia and metastatic seeding in hvKP infection allow earlier symptom onset, leading to earlier detection and thus smaller abscess size. Anatomically, the right lobe of the liver (segments V–VIII) is more frequently involved, possibly due to uneven drainage from the superior mesenteric vein into the portal system (19). Elevated lactate enhances hvKP virulence by promoting CPS biosynthesis: lactate downregulates the expression of IIA-D genes (gfrA, gfrB, gfrC, gfrD) in the mannose-specific phosphotransferase system (man-PTS), reducing intracellular cyclic adenosine monophosphate (cAMP) levels and decreasing the binding of cAMP to cAMP receptor protein (CRP), ultimately promoting CPS synthesis (18). Regrettably, lactate levels were not measured in this patient, which to some extent limited the comprehensive assessment of the clinical condition, but has also highlighted an essential measure that should be prioritized in subsequent clinical research and practice.

Management of IKPLAS requires individualized strategies based on disease severity, infection sites, and antimicrobial susceptibility. For diabetic patients, strict glycemic control is fundamental. Sufficient-dose and full-course antibiotic treatment should be administered early based on the results of drug susceptibility testing. Empirical antibiotic therapy typically includes third-generation cephalosporins or carbapenems for multidrug-resistant strains (20). The duration of treatment needs to be adjusted with reference to the remission of fever, the size of the liver abscess, and the neutrophil count (2). A typical regimen consists of 2–3 weeks of intravenous antibiotics followed by 2–4 weeks of oral therapy (16), though optimal duration and timing of intravenous-to-oral transition remain debated, which require more high-quality clinical studies to clarify. In addition to the aforementioned commonly used antibiotics, rifampicin has also shown certain potential in the treatment of IKPLAS. Studies have confirmed that rifampicin possesses anti-mucus activity and exerts a good inhibitory effect on hvKP. Clinical improvement with meropenem-rifampin combination therapy has been documented (21). For liver abscesses, management depends on size and liquefaction status. Lesions <3 cm or partially liquefied in the early stage may respond to antibiotics alone. Fully liquefied abscesses >3 cm typically necessitate image-guided percutaneous drainage for effective evacuation of purulent material and to promote recovery (22).

Despite its low incidence, IKPLAS is highly destructive and often initially asymptomatic, leading to delayed or missed diagnosis. Therefore, heightened awareness is crucial for early detection and improved outcomes. Clinicians should suspect IKPLAS in diabetic patients presenting with K. pneumoniae bacteremia, meningitis, or endophthalmitis, prompting liver imaging. Similarly, persistent fever despite appropriate antibiotic therapy in bacteremic patients warrants evaluation for occult abscesses.

Several important limitations of this study warrant acknowledgment. First, this single-case design carries inherent methodological constraints: the generalizability of our findings is inherently limited. Second, molecular confirmation of hvKP was not performed in this case. Third, the patient received multimodal therapy comprising systemic antibiotics, percutaneous liver abscess drainage, catheter-directed aspiration thrombectomy, and anticoagulation; as such, the independent contribution of each therapeutic component to the favorable clinical outcome cannot be disentangled or quantified. Additionally, several key clinical uncertainties remain unresolved by this report. The optimal timing of endovascular intervention for IKPLAS-related retrohepatic IVC bacterial embolus remains to be established. Furthermore, the optimal duration of antimicrobial therapy and the most appropriate anticoagulation strategy—including agent selection, dosing regimen, and total treatment course—for this specific clinical scenario remain poorly defined. Larger multicenter cohort studies and comparative clinical trials are warranted to address these knowledge gaps and establish standardized management protocols.

## 4 Conclusion

This case represents a detailed report of interventional treatment for bacterial embolism in the retrohepatic IVC secondary to IKPLAS. Given the highly invasive nature of hvKP and its propensity for hematogenous dissemination, clinicians must remain vigilant for bacterial embolism as a potential complication. In this patient, balloon dilation combined with catheter aspiration successfully restored IVC patency and prevented life-threatening complications such as pulmonary embolism. This interventional approach may serve as a valuable adjunct to standard antibiotic therapy in select cases of IKPLAS-related vascular complications. However, as a single-case report, our findings are limited by sample size. Future multicenter, large-scale studies are needed to validate the efficacy and safety of interventional therapy in IKPLAS-related IVC bacterial embolism, define optimal patient selection criteria, procedural timing, and perioperative management strategies, and ultimately establish higher-level evidence for standardized care.

## Data Availability

The raw data supporting the conclusions of this article will be made available by the authors, without undue reservation.
